# Circadian rhythms in the tumor microenvironment: spatiotemporal immune regulation and chronotherapeutic opportunities

**DOI:** 10.3389/fimmu.2026.1878388

**Published:** 2026-07-21

**Authors:** RenJie Zhao, YuXuan Xiao, XingXing Fan

**Affiliations:** Dr. Neher’s Biophysics Laboratory for Innovative Drug Discovery, State Key Laboratory of Mechanism and Quality of Chinese Medicine, Faculty of Chinese Medicine, Macau University of Science and Technology, Macau, China

**Keywords:** CAR-T therapy, chrono-pharmacology, circadian rhythm, immune checkpoint inhibitor, tumor microenvironment

## Abstract

The tumor microenvironment (TME) is increasingly recognized as a temporally organized ecosystem rather than a static structural niche. Circadian rhythms, generated by transcriptional–translational feedback loops involving CLOCK, BMAL1, PER, CRY, REV-ERB, and ROR, coordinate systemic physiology and local cellular programs that are directly relevant to tumor initiation, progression, and therapeutic response. In this review, we summarized how circadian regulation shapes tumor rhythmicity across multiple biological scales, from central clock-mediated synchronization to peripheral clocks within epithelial cells, stromal cells, adipocytes, and immune populations. Emphasis is placed on the spatiotemporal regulation of antitumor immunity within the TME. At the same time, dendritic cell migration, antigen presentation, CD8^+^ T cell infiltration, and T cell exhaustion display time-dependent features that influence the efficacy of immune surveillance and immunotherapy. These findings supported a four-dimensional view of the TME, in which biological timing is a critical determinant of immune competence. We further discussed emerging therapeutic strategies that exploit circadian biology, including small-molecule clock modulators, rhythm-responsive nanomedicine, chronologically optimized CAR-T cell therapy, and time-of-day-dependent immune checkpoint blockade. Although most mechanistic evidence remains preclinical, and many clinical observations are retrospective, current data suggest that treatment timing may be a modifiable, low-cost parameter for improving anti-tumor efficacy while reducing toxicity. Finally, we highlighted future opportunities in microbiome-informed chronotherapy, multi-omics profiling, and digital twin modeling. Integrating temporal information into oncology may shift precision medicine from a static biomarker-driven framework toward a dynamic, time-resolved therapeutic paradigm.

## 1 Circadian clock architecture as a biological framework for tumor rhythmicity

The mammalian circadian system is organized as a multilevel timing network that integrates environmental signals with endogenous physiological programs. At the organismal level, the suprachiasmatic nucleus (SCN) of the anterior hypothalamus functions as the principal circadian pacemaker (1). Light, the dominant external zeitgeber, is detected mainly by melanopsin-expressing intrinsically photosensitive retinal ganglion cells and transmitted to the SCN through the retinohypothalamic tract (2). Within SCN, photic input activates neurotransmitter-dependent signaling pathways, including glutamatergic and pituitary adenylate cyclase-activating polypeptide-mediated responses (3), which converge on calcium-sensitive transcriptional regulators such as cAMP response element-binding protein (CREB) (4). These signaling events reset the phase of clock gene expression and enable the SCN to align endogenous rhythms with the external light-dark cycle. Through neural projections, autonomic outflow, endocrine cues, body temperature oscillations, and behavioral rhythms such as feeding and sleep–wake cycles, the SCN coordinates peripheral clocks across metabolically diverse tissues, including the liver, adipose tissue, skeletal muscle, pancreas, heart, and lung. In this way, the central clock provides systemic temporal order while allowing tissue-specific oscillators to adapt circadian information to local physiological demands.

**Figure 1:**
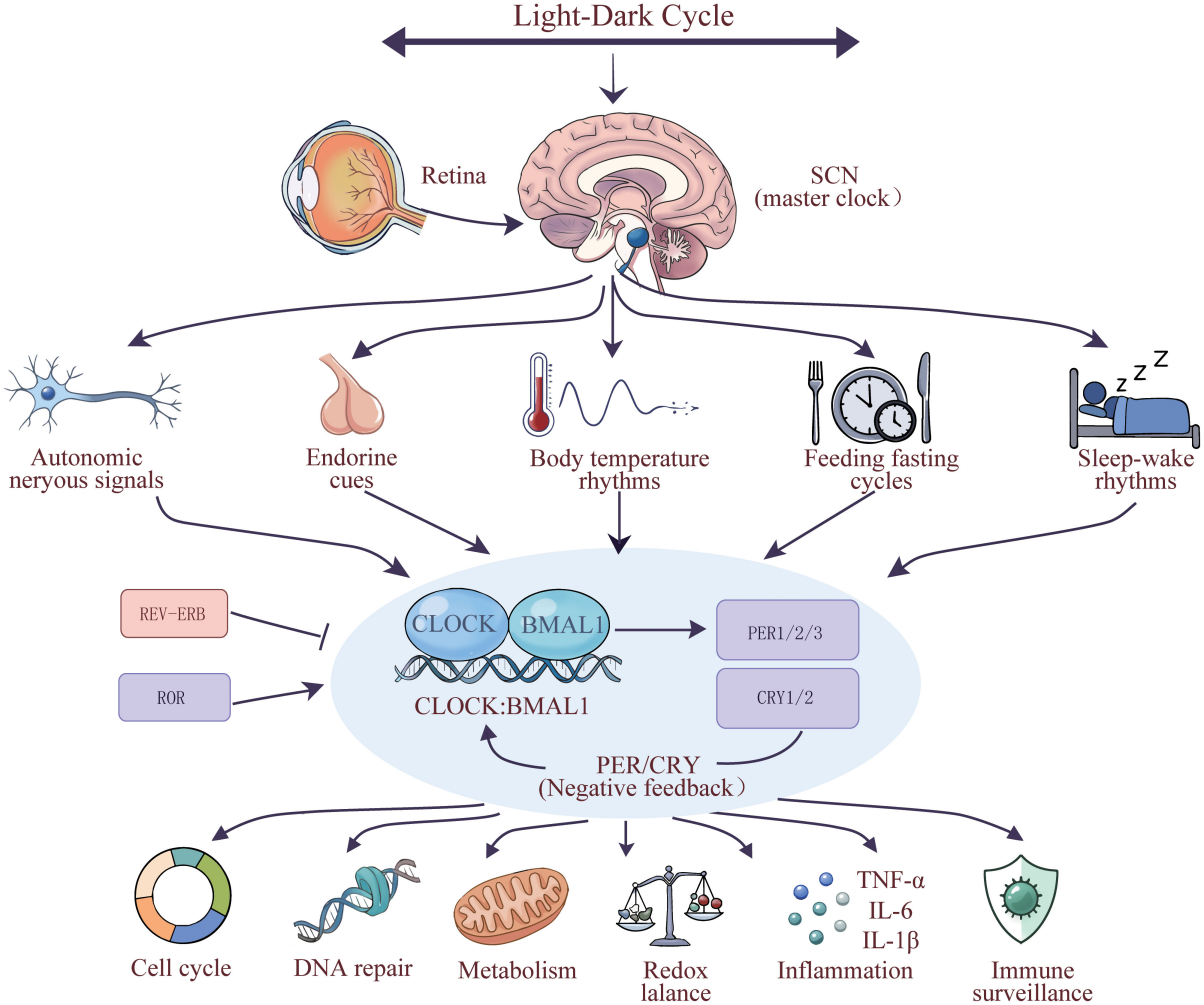
Rhythmic or disrupted clock signaling influences TME.

At the cellular level, circadian rhythmicity is generated by self-sustained molecular oscillators (**Figure 1**). The core of this timing machinery is the transcription–translation feedback loop. In the primary feedback loop, CLOCK and Brain and Muscle ARNT-Like 1 (BMAL1) form a transcriptionally active heterodimer that binds enhancer box (E-box) motifs in the regulatory regions of target genes and promotes the rhythmic transcription of Period genes, including PER1, PER2, and PER3, and Cryptochrome genes, including CRY1 and CRY2. After translation into the cytoplasm, PER and CRY proteins gradually accumulate and assemble into inhibitory complexes (5, 6). These complexes subsequently translocate into the nucleus, where they suppress CLOCK/BMAL1-dependent transcription. This delayed repression reduces new PER and CRY transcription, allowing the existing PER/CRY complexes to be degraded. The timing of this degradation is tightly controlled, particularly through casein kinase 1δ/ϵ (CK1δ/ϵ) mediated phosphorylation of PER proteins, which regulates their stability, turnover, and, consequently, the length of the circadian period (7). Once PER/CRY-mediated repression declines, CLOCK/BMAL1 activity is restored, initiating another round of transcription. This cyclical sequence of activation, delayed inhibition, degradation, and reactivation gives rise to near-24-hour molecular oscillations in individual cells.

The core CLOCK/BMAL1–PER/CRY loop is further stabilized by interlocking auxiliary feedback circuits, among which the REV-ERB/ROR axis is particularly important. CLOCK/BMAL1 drives the rhythmic expression of Rev-ERB and selected ROR genes. REV-ERBα and REV-ERBβ repress Bmal1 transcription by binding ROR response elements and recruiting transcriptional co-repressor complexes. In contrast, ROR proteins generally activate Bmal1 expression through the same or overlapping regulatory sites. The opposing actions of REV-ERBs and RORs generate a competitive regulatory module that fine-tunes the phase, amplitude, and robustness of the molecular clock. Because REV-ERBs and RORs belong to the nuclear receptor superfamily, this auxiliary circuit also links circadian timing to metabolic regulation, inflammatory signaling, and tissue-specific transcriptional programs (8). Thus, the circadian clock is not merely a timekeeping device but also a regulatory framework that converts environmental timing cues into cell- and tissue-specific biological outputs.

Clock-controlled genes largely mediate the physiological influence of the circadian clock. While core clock genes constitute the molecular timing apparatus, clock-controlled genes serve as the major effector layer, translating rhythmic information into functional cellular programs. By regulating these downstream targets temporally, the circadian system modulates processes essential for cellular homeostasis, including cell cycle progression, DNA damage sensing and repair, mitochondrial activity, redox balance, and metabolic flux (9). Among these outputs, metabolic regulation has been extensively characterized. Coordination between the central pacemaker and peripheral metabolic clocks contributes to daily rhythms in glucose homeostasis, endogenous glucose production, insulin sensitivity, lipid metabolism, and energy utilization (10). These rhythmic programs allow cells and tissues to anticipate predictable changes in nutrient availability and physiological demand, thereby improving metabolic efficiency and maintaining internal stability.

Disruption of this temporal organization can have broad pathological consequences. Chronic circadian misalignment caused by night shift work, irregular light exposure, persistent jet lag, or altered feeding–sleep schedules has been associated with metabolic dysfunction, impaired immune regulation, cardiovascular disease, and increased cancer susceptibility. In line with this evidence, night shift work involving circadian disruption has been classified by the International Agency for Research on Cancer as probably carcinogenic to humans (11). From an oncological perspective, this classification is especially relevant because many processes governed by the circadian clock, including cell proliferation, DNA repair, apoptosis, metabolism, immune surveillance, and inflammatory responses, are also central to tumor initiation and progression. Therefore, understanding the hierarchical organization of the circadian system, from SCN-mediated systemic synchronization to cell-autonomous transcriptional oscillators and clock-controlled homeostatic outputs, provides a necessary conceptual foundation for exploring how circadian dysregulation contributes to tumor biology.

## 2 Spatiotemporal dynamics of the tumor microenvironment

**Figure 2:**
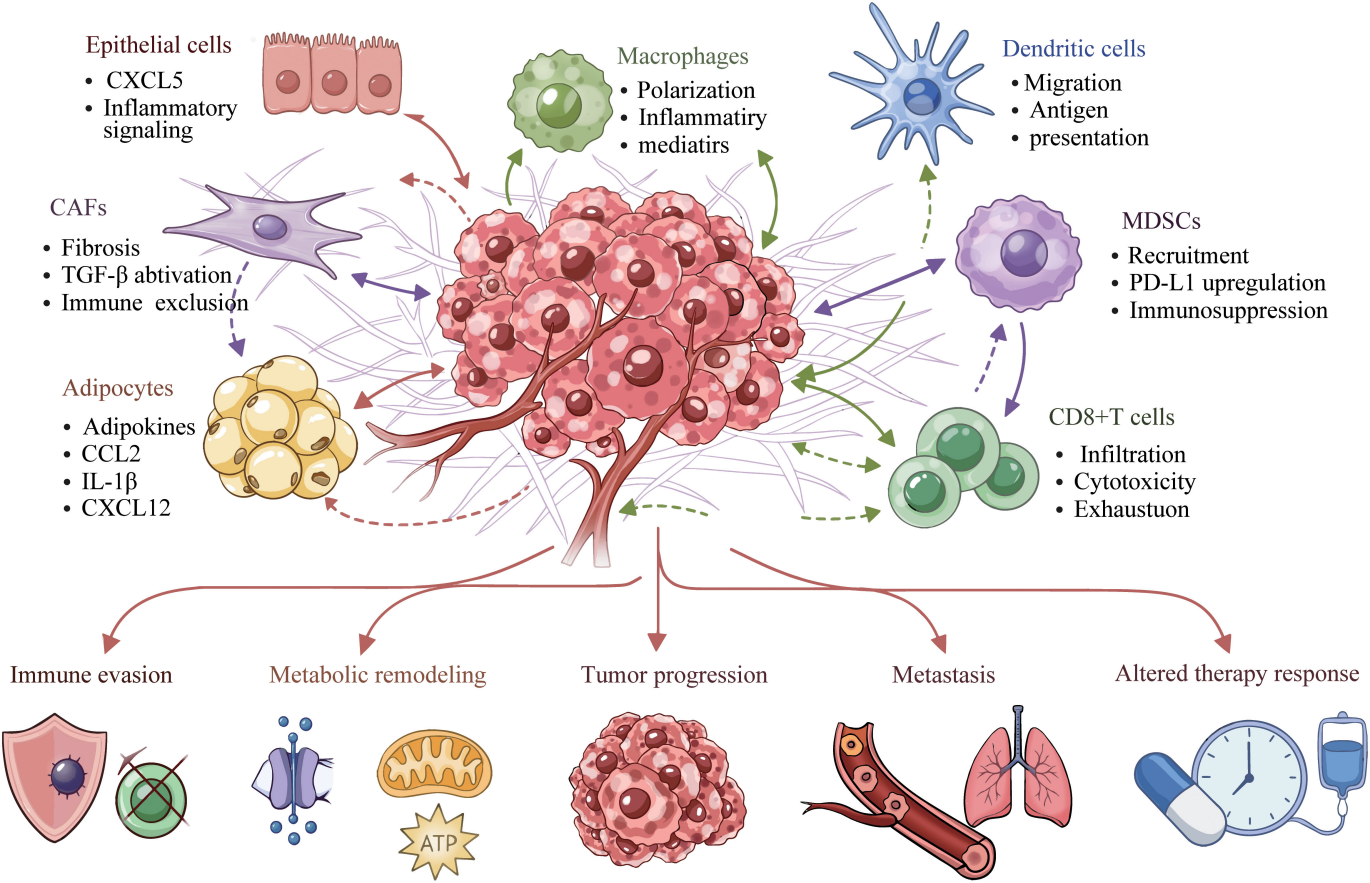
Summary of time-gated antitumor immunity in the TME.

The conceptualization of the TME has undergone a profound paradigm shift over the past decade, transitioning from a static, three-dimensional architectural model to a highly dynamic, four-dimensional chronobiological ecosystem (12, 13). Historically, the foundational understanding of the TME was largely restricted to spatial parameters, emphasizing the anatomical positioning of neoplastic cells, the density of the surrounding stromal populations, and the physical infiltration coordinates of diverse immune cohorts (14, 15). However, recent high-resolution investigations have irrefutably established that the immunological topography of the TME is profoundly non-stationary, operating instead as a fluid framework dictated by the molecular circadian clock (16, 17). This temporal dimensionality exerts rigorous, ubiquitous control over both the innate and adaptive arms of the mammalian immune system, systematically orchestrating diurnal oscillations in leukocyte trafficking, chemokine gradient secretion, and antigen cross-presentation efficiencies. The core circadian regulatory machinery, functioning primarily through the BMAL1/CLOCK heterodimeric transcription complex, actively and cyclically binds to canonical E-box elements situated within the promoter regions of critical immune-regulatory genes (12). Upon this rhythmic transactivation, downstream molecular cascades dictate the temporal capacity of the immune system to either recognize or ignore malignant transformations. Consequently, any pathological disruption of these intrinsic chronobiological networks, whether induced by environmental circadian misalignment, such as chronic shift work, or through localized genetic ablation within the epithelial compartment, precipitates a catastrophic breakdown of local immune homeostasis. Within the TME, this spatiotemporal dysregulation ultimately facilitates profound tumor immune evasion, fundamentally altering the trajectory of oncogenesis (16). This disruption manifests through distinct, time-dependent immunological phenomena, most notably the tidal, chemokine-driven accumulation of innate immunosuppressive populations and the rhythmic modulation of dendritic cell-mediated adaptive immune hubs (13). Therefore, this chapter meticulously delineates the spatiotemporal dynamics of the TIME, systematically transitioning from static spatial mapping to a comprehensive four-dimensional chronobiological paradigm. Specific and exhaustive focus will be directed toward the catastrophic, circadian-dependent recruitment of myeloid-derived suppressor cells (MDSCs) and the sophisticated time-gating theory governing the rhythmic efficacy of adaptive T cell responses (**Figure 2**).

### 2.1 Circadian regulation of epithelial, stromal, and adipose components

The structural and immunological integrity of the intestinal epithelial barrier is significantly connected to its circadian network function (18). This highly specialized epithelial clock serves as a primary, localized guardian against microenvironmental inflammatory pathogenesis and subsequent oncogenic transformation, functionally segregating the lumen from the underlying lamina propria (19). At the molecular apex of this complex regulatory network, Bmal1 operates as a core clock transcription factor (20). The rhythmic expression of Bmal1 coordinates a vast array of downstream transcriptomic targets that are essential for normative epithelial renewal, programmed cell death, and continuous immune epithelial crosstalk (21). Under physiological homeostatic conditions, the temporal expression profile of the Bmal1 transcript peaks robustly during the early resting phase of the organism and undergoes a strictly programmed attenuation during the early active phase (22). However, disruptions to this circadian rhythm structure, whether systemic or localized, can lead to severe, immediate microenvironmental imbalances. When the circadian clock is disrupted, the intestinal tissue is highly susceptible to cancer initiation and immunosuppressive reprogramming (23).

Researchers have successfully demonstrated the immunological consequences of disrupting the epithelial cell circadian rhythm using an advanced, genetically engineered mouse model of colorectal cancer (CRC). Specifically, researchers generated Bmal1^−/−^ and Apc^+/−^ mice. In the Bmal1^−/−^ mice, circadian clock function was selectively disrupted in intestinal epithelial cells while remaining intact in peripheral tissues. Disruption of the Bmal1 signaling pathway induced rapid pathological alterations in the intestinal epithelium, characterized by excessive epithelial cell proliferation and marked dysregulation of the temporal secretion patterns of pro-inflammatory cytokines. These changes ultimately promoted the rapid establishment of a highly immunosuppressive TME (16).

Beyond the epithelial barrier of the TME, chronobiological regulation also influences stromal and adipose components. Among these, cancer-associated fibroblasts (CAFs), a major stromal cell population, are tightly controlled by intrinsic molecular clock signaling. Studies have shown that the absence of the Bmal1 gene in the host matrix exacerbates the fibrotic phenotype and accelerates tumor metastasis. Mechanistically, the circadian clock gene Bmal1 inhibits plasmin production by transcriptionally targeting plasminogen activator 1 (PAI-1), thereby suppressing transforming growth factor beta (TGF‐*β*) function. In mice, Bmal1 knockout decreases PAI-1 expression in the TME, which, in turn, upregulates tissue plasminogen activator (tPA) and urokinase plasminogen activator (uPA) to activate plasmin. Subsequently, plasmin activates TGF-β, inducing tumor fibrosis and transforming CAFs into myofibroblastic cancer-associated fibroblasts (myoCAF). These processes collectively promote tumor growth and metastasis (24). The fibrotic barrier formed by these TGF-β-activated myoCAFs not only physically excludes cytotoxic T cells from the tumor but also facilitates the recruitment of immunosuppressive innate immune cell populations, including N2 neutrophils and M2 polarized macrophages (25). Tumor-associated adipocytes (TAAs) are another important part of the TME’s rhythm regulatory system. The secretion of adipocyte metabolic factors, adipokines, and inflammatory cytokines is regulated by the Clock/Bmal1 heterodimer, thereby coordinating energy homeostasis. Disruption of the TAAs rhythm triggers chronic low-grade adipose tissue inflammation. This inflammatory state prompts the TAAs to secrete chemokines, including CCL2, IL-1β, and CXCL12. These factors actively recruit systemic macrophages to the TME and induce their polarization towards M2 macrophages (26). Furthermore, in this dysregulated adipose microenvironment, recruited MDSCs exhibit significant upregulation of the immune checkpoint molecule Programmed Death-Ligand 1 (PD-L1), exacerbating the local immunosuppressive burden (27). Disruption of the circadian regulator Bmal1 in intestinal epithelial, stromal, and adipose compartments promotes an inflammatory imbalance, remodeling of the immunosuppressive microenvironment, fibrosis, and tumor progression through coordinated alterations in epithelial homeostasis, CAF activation, and immune cell polarization.

### 2.2 MDSCs recruitment and immunosuppressive remodeling

The mechanistic link between local rhythm disturbances and the establishment of a strong innate immunosuppressive barrier is mediated by abnormal rhythmic secretion of pro-inflammatory chemokines by MDSCs. Under physiological conditions, intrinsic rhythmic mechanisms actively inhibit carcinogenic signaling cascades, such as the classic Wnt/β-catenin pathway (16). When key rhythm regulators (such as Bmal1) are disrupted, this rhythm regulation mechanism fails, leading to the activation of downstream proto-oncogenes, especially c-Myc (28). This increase in oncogenes leads to high expression of the pro-inflammatory chemokine CXCL5 in epithelial cells. Subsequently, CXCL5 is secreted from the tumor core and radiates outward into the systemic circulation, actively recruiting CXCR2-expressing MDSCs from the bone marrow into the TME (29). Rather than maintaining a static equilibrium, these recruited granulocytic myeloid populations differentiate into highly immunosuppressive effectors that exhibit exceptionally high PD-L1 expression and undergo circadian oscillations within the tumor stroma (30). Once recruited into the TME, MDSCs do not merely accumulate but actively remodel local immune signaling networks through multiple complementary mechanisms. In addition to expressing high levels of PD-L1, MDSCs suppress T-cell activation by producing arginase-1 (ARG1), inducible nitric oxide synthase (iNOS), and reactive oxygen species (ROS), leading to L-arginine depletion, impaired T-cell receptor signaling, and reduced cytotoxic function (31). These suppressive pathways act in concert with circadian-regulated chemokine signaling to establish an immunosuppressive microenvironment.

Importantly, these rhythmic fluctuations appear to influence not only MDSC abundance but also their suppressive activity, suggesting that both immune-cell trafficking and functional polarization may be subject to circadian regulation, although the molecular mechanisms governing these temporal changes remain incompletely understood. High-resolution *in vivo* tracking has revealed that in hosts with intact systemic clocks, the absolute abundance of these highly suppressive PD-L1^+^ MDSCs peaks decisively during the early active phase (Zeitgeber Time 16, ZT16) and plummets to a distinct nadir during the early rest phase (ZT4) (16, 17). Furthermore, recent evidence suggests that rhythm disturbances promote the accumulation of MDSCs in distant organs, thereby accelerating CRC metastasis. This process is primarily mediated by gut microbiota metabolites, such as taurine (32). This dynamic phenomenon highlights a complex, multi-layered regulatory structure: local epithelial rhythm disruption initiates chronic CXCL5 homing signals, microbial metabolites support systemic survival. In contrast, overall systemic rhythms impose a strict time-gating mechanism that determines the eventual rhythmic infiltration of these myeloid cells into tissues. Consequently, rhythmic accumulation of MDSCs may contribute to time-dependent suppression of antitumor immunity through coordinated inhibition of CD8^+^ T-cell activation, reduced antigen presentation, and impaired dendritic-cell function. However, the relative contributions of each suppressive mechanism are likely to vary across tumor types and remain an active area of investigation.

### 2.3 Dendritic cells trafficking and adaptive antitumor immunity

Although increasing evidence indicates that adaptive anti-tumor immunity is influenced by circadian regulation, the magnitude and temporal characteristics of these effects appear to vary across experimental models, tumor types, and biological contexts. As typical antigen-presenting cells, DCs constitute key immune synapses connecting innate immune surveillance and adaptive T-cell cytotoxicity (33). These cells metastasize from peripheral tumors to tumor-draining lymph nodes (dLNs), the primary site of adaptive immune initiation, and occur at a non-constant rate (34). In a mouse melanoma model, tumor transplantation performed late afternoon (ZT9) resulted in a significant reduction in final tumor volume compared to nighttime transplantation. This difference was primarily attributed to the accumulation of tumor-derived antigens presented by a large subset of migratory DCs (CD103^+^ CD11c^+^ MHCII^high^) in dLNs (17). Crucially, this significant difference in antigen-presenting capacity was independent of the biochemical rate of intracellular antigen processing, indicating that dynamic physical transport regulates circadian rhythmic immune function. Migrating DCs provide the necessary spatial basis for the physiological inflow into dLNs, but a co-stimulatory signal is required for successful antigen cross-presentation to CD8^+^ T cells (35). High-resolution single-cell RNA sequencing revealed that the key co-stimulatory molecule CD80 was highly enriched in the DC “morning cluster” (peaking at ZT9). The Bmal1 protein exhibits highly rhythmic, phase-locked binding kinetics to a typical E-box precisely located within the proximal promoter region of the CD80 gene, thereby systematically activating its transcription. In the absence of a complete DC intrinsic clock, this characteristic rhythmic oscillation of CD80 is lost, markedly reducing DCs’ ability to transmit rhythm-dependent co-stimulatory signals to interacting T cells (17). Nevertheless, the extent to which DC-intrinsic clock regulation contributes to immune activation relative to systemic neuroendocrine cues or tumor-specific microenvironmental factors remains an area of active investigation.

Collectively, these findings have led to the proposal of a circadian “time-gating” model, in which adaptive immune activation may be optimized during specific temporal windows when dendritic cell migration, antigen presentation, and co-stimulatory signaling are synchronized. Although this conceptual framework is supported by several preclinical studies, the timing, magnitude, and underlying mechanisms of these rhythmic immune responses vary across experimental systems. Moreover, direct evidence from human studies remains limited, and additional investigations are required to determine whether similar temporal immune windows exist across different tumor types and clinical settings. Therefore, the time-gating hypothesis should currently be viewed as a promising conceptual framework for guiding future chrono-immunotherapy research rather than as a universally established biological principle.

### 2.4 CD8^+^ T cell trafficking and exhaustion

#### 2.4.1 Diurnal infiltration and the SMAD7-CXCR4 axis

The potency of the adaptive immune response does not depend solely on the temporal dynamics of DCs within lymph nodes, but also on the ability of CD8^+^ T cells to infiltrate the solid TME rhythmically. Recent studies have revealed the rhythmic infiltration pattern of CD8^+^ T cells. The anti-tumor function of CD8^+^ T cells in the TME is significantly regulated by circadian rhythms, driven by the cyclical interaction between constant TGF-β signaling and the inhibitory protein SMAD7. This interaction induces a pronounced daily oscillation in the expression of CXCR4 receptors on T cell surfaces. During periods of peak CXCR4 expression, T cells are strongly recruited by CXCL12 continuously released from surrounding CAFs. As a result, cytotoxic T cells are physically drawn away from the malignant tumor core and sequestered in CAF-rich zones, effectively trapping them and restricting their ability to engage and destroy cancer cells (13).

#### 2.4.2 Epigenetic-metabolic crosstalk governing CD8^+^ T cell exhaustion switch

Beyond the physical entrapment orchestrated by the stroma, the sustained effector function of CD8^+^ T cells is compromised by prolonged antigen exposure and intense nutritional competition within the TME. Recent breakthrough research appears to redefine T cell exhaustion, shifting it from a mere consequence of surface receptor signaling to a regulated epigenetic cell fate driven by pathological metabolic reprogramming (36). During the initial effector state maintenance phase, highly functional CD8^+^ T cells predominantly utilize exogenous acetate as a primary metabolic substrate (37). To achieve this, effector T cells rely on acetyl-CoA synthase 2 (ACSS2) to metabolize acetic acid to the universal acetyl donor acetyl-CoA. The acetyl-CoA is specifically directed to the p300 histone acetyltransferase to form a functional ACSS2-p300 protein complex. The ACSS2-p300 complex is essential for maintaining stable histone acetylation at key immune-killing and memory gene loci, thereby sustaining robust anti-tumor cytotoxicity (38).

However, in response to chronic stimulation, T cells progressively transition into a terminally exhausted state, undergoing profound metabolic reprogramming characterized by the severe downregulation of ACSS2 and an enhanced reliance on citrate metabolism (39). During this ATP-citrate lyase-dominated phase, exhausted T cells utilize ATP-citrate lyase (ACLY) to synthesize essential acetyl-CoA from citrate rather than acetate. This pathological shift in nutrient preference drives the subcellular spatial re-localization of ACLY, facilitating its specific interaction with the histone acetyltransferase KAT2A. The newly assembled ACLY-KAT2A complex becomes aberrantly enriched at the *cis*-regulatory elements of exhaustion-associated genes, directly mediating histone acetylation and consequently hyperactivating the transcription of inhibitory immune checkpoints, including PD-1 and TIM-3 (40). This metabolic transition from acetate to citrate is associated with extensive epigenetic remodeling that reinforces exhaustion-associated transcriptional programs and promotes the progression toward a terminally exhausted phenotype (41).

From a chronobiological perspective, circadian fluctuations in nutrient availability within the TME may influence the metabolic environment experienced by infiltrating CD8^+^ T cells. It is therefore conceivable that temporal mismatches between T-cell metabolic demand and local nutrient availability could contribute to metabolic remodeling associated with exhaustion. However, direct experimental evidence demonstrating that circadian metabolic oscillations directly regulate the ACSS2–ACLY metabolic switch remains limited, and this proposed link should currently be regarded as a conceptual framework requiring further experimental validation.

### 2.5 Neural regulation as an emerging interface between circadian rhythms and tumor immunity

Accumulating evidence suggests that circadian regulation of the TME extends beyond immune and stromal compartments to include dynamic interactions with the peripheral nervous system. Increasing recognition of neuroimmune communication has revealed that neural signaling not only influences tumor growth and metastasis but also shapes immune-cell recruitment, inflammatory responses, and tissue remodeling within the TME (42).

Recent studies further demonstrate that bidirectional communication exists between tumors and the nervous system. For example, tumor-derived extracellular vesicles have been shown to induce neuronal necroptosis by transferring O-GlcNAcase, thereby promoting perineural invasion and remodeling of the local neural microenvironment. These findings highlight that tumors actively reshape neural networks rather than simply responding to neural inputs (43).

From a chronobiological perspective, this emerging neuroimmune axis provides an additional layer through which circadian rhythms may influence tumor immunity. Circadian regulation of sympathetic activity, neuroendocrine signaling, and peripheral nerve function may indirectly affect immune-cell trafficking, cytokine production, and stromal activation. Nevertheless, direct experimental evidence linking circadian neural regulation to tumor-immune remodeling remains limited, and the precise mechanisms by which neural rhythms coordinate immune responses within the TME require further investigation. Thus, the nervous system should be considered an emerging component of the circadian tumor ecosystem and an important direction for future chrono-oncology research.

## 3 Mechanisms of pharmacological circadian rhythm regulation and their medical applications in the TME

**Figure 3:**
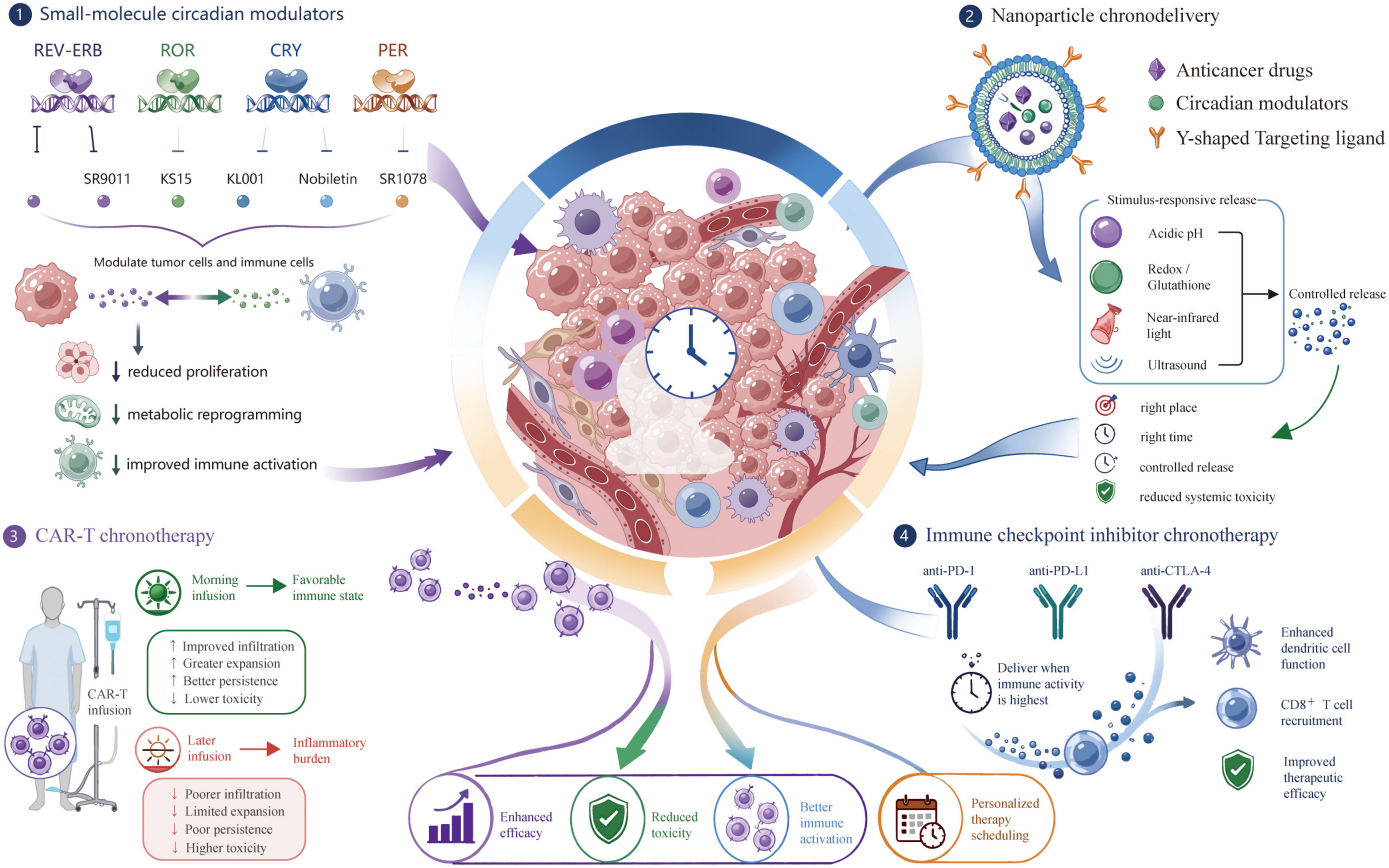
Chronotherapeutic strategies targeting tumor rhythmicity.

Tumors are increasingly recognized as dynamic biological systems in which cellular metabolism, proliferation, immune surveillance, and stromal interactions are shaped not only by spatial heterogeneity but also by temporal regulation (44). At the molecular level, circadian rhythms are generated by feedback loops centered on CLOCK, Bmal1, PER1/2/3, and CRY1/2, with auxiliary regulation by the nuclear receptors REV-ERB and ROR (45). Disruptions in these rhythm networks are associated with the development of various tumors, metabolic reprogramming, altered DNA damage responses, and impaired anti-tumor immunity (46). Therefore, chrono-pharmacology has become an important framework for cancer research, as the efficacy and toxicity of anticancer therapies can vary with the timing of administration and the circadian rhythms of the host and TME (**Figure 3**).

### 3.1 Small-molecule therapeutics targeting the circadian TME

Drug therapies targeting circadian rhythm components have attracted increasing attention as a strategy to remodel tumor cell adaptation and the TME simultaneously. Current circadian-targeting small molecules can be broadly classified according to their modulation of distinct clock components, including REV-ERBs, CRYs, PERs, and RORs. Although these agents differ in their molecular targets, they share a common therapeutic rationale of restoring circadian homeostasis to influence tumor metabolism, immune regulation, and cellular stress responses within the TME (47). Among the rhythm-regulating compounds currently under investigation, synthetic REV-ERB agonists, such as SR9009 and SR9011, have shown antitumor activity in preclinical models (48). REV-ERB activation inhibits tumor cell survival by reprogramming metabolic pathways and stress adaptation procedures. However, the specific mechanisms of action of its downstream effector molecules may vary depending on tumor type. This is particularly important in the TME, where metabolic competition, hypoxia, and nutrient restriction amplify the functional consequences of circadian rhythm disruption (49). Additional REV-ERB ligands, such as GSK4112 (SR6542) and GSK2945, with improved receptor selectivity, have also demonstrated anti-tumor efficacy in hematologic and solid tumor models, further supporting this therapeutic concept (50).

Beyond direct effects on tumor cells, several circadian modulators have also been reported to reshape immune components of the TME, particularly macrophage polarization and inflammatory cytokine production. These findings suggest that circadian intervention may simultaneously influence both malignant cells and their surrounding immune niche (51). These small molecules promote local transformation by targeting TAM’s circadian rhythm to regulate polarization switching, thereby reducing immune tolerance. They have significant potential to synergize with established immunotherapies to improve clinical outcomes.

Conversely, pharmacological agents targeting the Cryptochrome (CRY) and Period (PER) proteins also manifest significant context-dependent anti-tumor properties by manipulating the primary circadian feedback loop (52). The small-molecule inhibitor KS15, which specifically disrupts the structural interaction between CRY and BMAL1, has been shown to suppress tumor proliferation and dramatically enhance chemosensitivity in therapy-resistant breast cancer models (53). This indicates that precise uncoupling of circadian clock protein complexes can disrupt the time-metabolic shielding mechanism used by drug-resistant tumors to defend against cytotoxic damage. On the other hand, KL001 acts as a highly specific CRY activator and stabilizer, documented to prolong the circadian period, reduce overall amplitude, and suppress the rapid proliferation of glioblastoma stem cells (GSCs) (54). Similarly, natural and synthetic ROR agonists, such as the flavonoid Nobiletin and the synthetic compound SR1078, significantly enhance BMAL1 expression (55). Nobiletin administration directly inhibits the c-Myc oncogene-mediated signaling pathway, thereby inducing definitive cell cycle arrest during G1/S transition in various gastrointestinal and hepatocellular carcinoma lineages (56).

Collectively, current evidence indicates that circadian-targeting small molecules have the potential to simultaneously regulate tumor-intrinsic pathways and the immune microenvironment, providing a compelling rationale for chronotherapeutic intervention. However, most available evidence remains confined to *in vitro* systems and preclinical animal models, with relatively few studies addressing pharmacokinetics, optimal dosing schedules, long-term safety, or interactions with existing cancer therapies (57). Moreover, considerable heterogeneity in circadian organization across tumor types and individual patients may substantially influence therapeutic efficacy. Therefore, before these compounds can be translated into clinical practice, future studies should prioritize biomarker-guided patient stratification, treatment-timing optimization, and well-designed clinical trials to establish their safety and therapeutic benefit.

### 3.2 Nanomedicine-based rhythm-responsive drug delivery systems

A major obstacle to effective chronotherapy is that conventional drug delivery rarely maintains tissue drug exposure in synchrony with the temporal vulnerability of the TME. As a result, the expected time-dependent therapeutic advantage is often not achieved. In recent years, researchers have begun combining nanotechnology with circadian biology to address both spatial and temporal limitations. Compared with traditional formulations, nanocarriers can be engineered to respond to specific stimuli in the TME, allowing more controlled drug release. Some studies suggest that these systems may also be adjusted to better fit circadian variations in tumor metabolism and host physiology (58). More importantly, rhythm-responsive nanocarriers provide a potential platform for synchronizing drug delivery with circadian immune activity within the TME, thereby enhancing the efficacy of immunomodulatory therapies rather than merely improving pharmacokinetic performance.

Importantly, immune-cell trafficking, cytokine secretion, antigen presentation, and responsiveness to immune checkpoint blockade all exhibit circadian variation. Nanocarrier systems capable of temporally controlled drug release may provide an effective strategy to synchronize immunomodulatory interventions with periods of maximal immune competence (59). Therefore, rhythm-responsive delivery systems should be considered as enabling technologies that facilitate circadian immune regulation rather than independent therapeutic modalities.

One commonly used strategy is based on TME’s acidic nature. Due to enhanced glycolysis, tumor tissues usually maintain a slightly acidic extracellular pH (6.5-6.8), which can serve as a trigger for drug release (60). Interestingly, this acidic environment is not only a metabolic feature but may also interfere with circadian regulation in immune cells. For example, macrophages exposed to acidic conditions tend to lose their normal rhythmic activity and shift toward an immunosuppressive phenotype (61). Based on this, some nanocarriers have been designed to co-deliver anticancer drugs and clock-regulating molecules, aiming to partially restore immune function in the TME (46). Because oxidative stress also regulates immune-cell activation and circadian signaling, redox-responsive nanocarriers may further facilitate temporally coordinated immune modulation in addition to improving drug selectivity.

External stimuli, including near-infrared irradiation and ultrasound, further expand opportunities for temporally controlled drug administration. Beyond improving release precision, these approaches may permit synchronization of immunomodulatory therapies with periods of enhanced antigen presentation, T cell activation, or immune checkpoint responsiveness, although experimental evidence remains limited (62).

Overall, rhythm-responsive nanomedicine should be viewed not simply as a drug delivery technology but as a potential enabling platform for chrono-immunotherapy. By coordinating the spatial delivery of therapeutic agents with the temporal dynamics of immune regulation in the TME, these systems may improve immune activation while minimizing off-target toxicity. Nevertheless, current evidence remains largely preclinical, and whether circadian-guided nanomedicine can consistently enhance immunotherapeutic efficacy in patients remains to be established.

### 3.3 Temporal regulation and rhythmic dynamics in chimeric antigen receptor T-cell therapy

Adoptive cell therapies, particularly CAR-T cell approaches, have produced durable responses in hematologic malignancies. However, their application in solid tumors remains constrained by the immunosuppressive TME and physical barriers such as dense extracellular matrix deposition. Increasing evidence indicates that the functional performance of CAR-T cells—including persistence, expansion, and tumor infiltration—is not constant over time but is instead modulated by the host circadian system (63). Rhythms coordinate systemic immune processes through neuroendocrine signaling and local cytokine regulation, thereby shaping leukocyte trafficking, T-cell activation states, and inflammatory tone within the TME (64, 65). Consequently, the timing of CAR-T cell infusion has emerged as a potentially critical determinant of therapeutic efficacy.

A large retrospective study of patients with B-cell lymphoma demonstrated that treatment outcomes varied significantly by infusion time (66). Patients receiving CAR-T cells in the morning had higher complete response rates and overall survival than those treated later in the day. Notably, each hour of delay in administration was associated with a measurable increase in short-term mortality risk and treatment-related toxicity, including immune effector cell-associated neurotoxicity syndrome (ICANS) (66, 67).

Mechanistically, this time-of-day dependency is more likely to reflect coordinated circadian variation across multiple components of the TME rather than oscillation of the TME as a single entity. These include rhythmic changes in chemokine gradients, immune-cell trafficking, cytokine production, vascular function, stromal metabolism, and nutrient availability, all of which collectively influence CAR-T cell infiltration and activity. Experimental evidence suggests that cytotoxic T cell activity and tumor infiltration capacity exhibit circadian variation, with peak functionality occurring during specific phases of the light–dark cycle (68). Administering CAR-T cells during these optimal windows may enhance their ability to traverse tumor vasculature and facilitate migration through stromal barriers. In contrast, late-day infusion often coincides with elevated systemic inflammatory mediators, including interleukin-6 and acute-phase proteins, which can amplify cytokine release and toxicity (69). This pro-inflammatory milieu may impair CAR-T expansion while exacerbating adverse events, thereby reducing the therapeutic index.

At the cellular level, circadian regulation of metabolism further contributes to these effects. T cell bioenergetics, including glycolysis, oxidative phosphorylation, and mitochondrial function, follow daily oscillatory patterns governed by the clock gene. Enzymes such as isocitrate dehydrogenase are implicated in maintaining metabolic fitness and epigenetic stability in CAR-T cells, thereby potentially contributing to persistence and functional fitness (63). Thus, infusion during a biologically unfavorable phase may impose a dual burden: reduced intrinsic cellular fitness and a hostile tumor environment.

Current evidence suggests that circadian timing is a potentially modifiable variable that influences CAR-T cell efficacy and toxicity. Nevertheless, a clear distinction should be made between the robust mechanistic evidence supporting circadian regulation of immune-cell function and the comparatively limited clinical evidence available to date. Most mechanistic insights have been derived from experimental models, whereas human studies remain largely retrospective and heterogeneous. Moreover, substantial interindividual variation in chronotype, concomitant medications, comorbidities, environmental influences, and differences between experimental models and human physiology may all affect the clinical applicability of chronotherapeutic strategies. Importantly, implementing time-optimized CAR-T infusion schedules may also impose logistical challenges related to staffing, patient scheduling, and institutional workflows, particularly when biologically favorable treatment windows occur outside routine clinical hours. Therefore, although circadian-guided treatment represents a promising avenue for improving CAR-T therapy, prospective controlled clinical trials are required to determine whether the observed time-of-day associations reflect true circadian mechanisms or residual clinical confounding before standardized chronotherapeutic protocols can be recommended.

### 3.4 The clinical paradox of immune checkpoint inhibitor timing and microenvironmental mechanisms

ICIs, particularly those targeting PD-1/PD-L1 and CTLA-4 pathways, have transformed cancer therapy by restoring anti-tumor immunity by reversing T cell exhaustion and immune suppression within the TME (70, 71). Despite this success, clinical outcomes remain highly heterogeneous, with a substantial proportion of patients failing to respond even when tumors exhibit high PD-L1 expression (72). This discrepancy highlights a fundamental limitation of conventional biomarker strategies, which rely on static measurements and fail to capture the TME’s dynamic, temporal nature.

Recent retrospective clinical studies have reported associations between the timing of ICI administration and therapeutic outcomes, with several cohorts suggesting improved overall and progression-free survival following morning administration compared with later treatment (73). These observations support the hypothesis that circadian biology may contribute to variability in immunotherapy responses, although causality has not yet been established. Mechanistically, this temporal dependency is largely driven by circadian regulation of immune cell dynamics within the TME. DCs and tumor-associated macrophages exhibit rhythmic expression of chemokines and immune checkpoint molecules, including oscillations in PD-1/PD-L1 signaling. These oscillations coordinate the recruitment and activation of cytotoxic CD8^+^ T cells, resulting in time-of-day–dependent variations in immune infiltration (16). Administering ICIs during these peak immune-active phases may improve therapeutic responsiveness by aligning pharmacological blockade with the maximal presence of effector cells. Conversely, treatment during circadian troughs may reduce efficacy due to insufficient immune cell engagement.

Collectively, current evidence suggests that circadian timing represents a promising avenue for optimizing immune checkpoint blockade. However, it is important to distinguish encouraging biological observations from clinically validated treatment strategies. Most available clinical evidence has been derived from retrospective analyses, whereas prospective randomized trials evaluating treatment timing remain scarce. Furthermore, differences in patient populations, tumor types, treatment schedules, and study design complicate comparisons across existing studies. Consequently, although chrono-immunotherapy has considerable translational potential, its incorporation into routine clinical practice will require prospective validation, standardized timing protocols, and careful evaluation of feasibility within contemporary oncology workflows.

### 3.5 Current challenges for clinical translation

Despite the compelling mechanistic evidence supporting circadian regulation of immune cell trafficking, antigen presentation, cytokine signaling, and therapeutic responsiveness within the TME, substantial challenges remain before these findings can be translated into standardized clinical practice. At present, most mechanistic insights have been generated from *in vitro* systems and preclinical animal models, whereas prospective clinical evidence remains comparatively limited. Although retrospective clinical studies have reported encouraging associations between treatment timing and therapeutic outcomes, prospective randomized clinical trials remain scarce. Consequently, the current evidence base is insufficient to establish standardized chrono-oncology protocols in routine clinical practice.

An additional challenge arises from the considerable interindividual variability in circadian biology. Differences in chronotype, age, sleep-wake behavior, comorbidities, concomitant medications, and environmental factors such as light exposure and feeding schedules may all influence circadian phase and immune function. Furthermore, circadian organization in experimental animal models differs substantially from that in humans, particularly with respect to activity patterns and metabolic rhythms, complicating the extrapolation of preclinical findings to clinical settings. These biological sources of heterogeneity highlight the need for individualized approaches rather than universal treatment schedules.

Practical implementation also presents important challenges. Coordinating treatment administration with individualized circadian phases may require modifications to hospital scheduling, infusion center operations, staffing patterns, and manufacturing workflows, particularly for complex cellular therapies such as CAR-T cells. Therefore, although chrono-oncology does not necessarily require new therapeutic agents, successful implementation may still incur substantial logistical and organizational costs.

Future studies should prioritize multicenter prospective randomized clinical trials incorporating circadian biomarkers, chronotype stratification, and standardized timing protocols. Such studies will be essential for distinguishing true circadian effects from potential confounding variables and for determining whether chronotherapeutic strategies can be consistently translated into routine oncology practice.

### 3.6 Future translational perspectives

**Figure 4:**
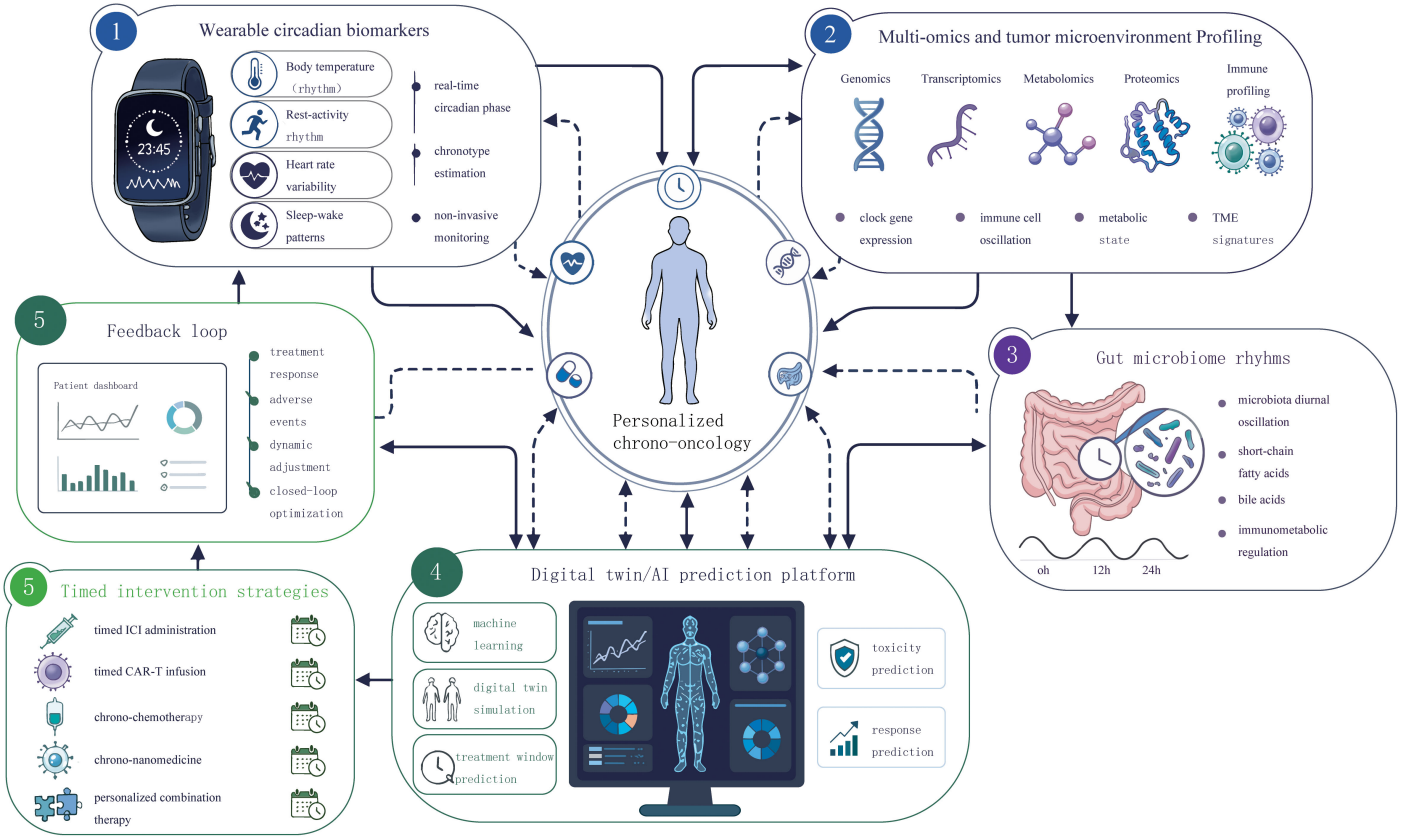
A future framework for personalized chrono oncology.

Although substantial progress has been made in elucidating the biological basis of circadian regulation within the TME, significant translational challenges remain before these discoveries can be broadly implemented in clinical oncology. Against this background, several emerging technologies may facilitate the future development of precision chrono-immunotherapy (**Figure 4**).

Recent advances in digital health technologies provide a feasible pathway toward this goal. Wearable biometric monitoring devices enable continuous, non-invasive acquisition of physiological parameters, including core body temperature, rest–activity cycles, and heart rate variability, which collectively serve as high-resolution proxies of endogenous circadian phase (74). Integration of these longitudinal datasets with machine learning algorithms enables the reconstruction of individualized circadian profiles and the prediction of optimal therapeutic windows for drug delivery. Such approaches may enable synchronization of immunotherapy with peak immune activation states, thereby enhancing treatment efficacy.

In parallel with biometric monitoring, the concept of “digital twins” is emerging as a transformative computational framework in oncology. Digital twins are patient-specific, multi-scale in silico models that integrate genomic, physiological, and microenvironmental data to simulate disease progression and treatment response (75). In the context of chrono-immunotherapy, these models can incorporate circadian oscillations in immune cell trafficking, chemokine gradients, and stromal properties, enabling prediction of temporally optimized dosing schedules (76). Importantly, digital twins enable iterative in silico testing of therapeutic strategies, reducing clinical risk while facilitating personalized treatment design.

Another rapidly expanding area is the interplay between circadian rhythms and the gut microbiome. The microbiota exhibits intrinsic diurnal oscillations that regulate the systemic availability of metabolites, such as short-chain fatty acids and bile acids, which, in turn, modulate immune responses and TME composition (77). Disruption of host–microbiome circadian alignment has been associated with impaired immunotherapy response and increased tumor progression. Consequently, microbiome-targeted interventions, including engineered probiotics and timed dietary modulation, are being explored as adjuvant strategies to enhance the efficacy of chrono immunotherapy.

From a therapeutic development perspective, integrating circadian biology into drug design is also gaining traction. Chrono-pharmacological strategies aim to align drug pharmacokinetics and pharmacodynamics with endogenous biological rhythms to maximize efficacy while minimizing toxicity (78). This is particularly relevant for immunotherapies, where circadian-dependent variations in immune activation and cytokine release can significantly influence both therapeutic outcomes and adverse events.

Looking forward, the convergence of multi-omics profiling, wearable-derived circadian biomarkers, microbiome analytics, and AI-driven modeling provides an encouraging framework for the future development of chrono-oncology. Nevertheless, these technologies remain largely investigational, and their clinical utility will require rigorous validation through prospective multicenter studies before widespread implementation can be recommended. Continued integration of biological, technological, and clinical evidence will be essential for translating chronobiological insights into safe, practical, and effective precision cancer therapies.

## 4 Conclusions

In summary, the TME should be redefined as a circadian-governed, spatiotemporally dynamic ecosystem, rather than a static structural entity. Circadian disruption acts as a central driver, synchronizing immunosuppressive cell recruitment, impairing antigen presentation, and accelerating CD8^+^ T cell exhaustion through epigenetic metabolic reprogramming. Critically, immunometabolic exhaustion emerges not merely as a downstream consequence, but as a time-encoded fate decision, linking metabolic substrate availability to irreversible epigenetic remodeling.

These insights collectively point to a necessary paradigm shift: time is not a variable in cancer therapy, but a therapeutic axis. Leveraging circadian biology through chrono-pharmacology, rhythm-responsive delivery systems, and temporally optimized immunotherapy may unlock durable responses that current strategies fail to achieve. Ultimately, integrating temporal precision into oncology is not optional, but essential for overcoming immune resistance and advancing toward sustained cancer control.

## References

[1] Hastings MH Maywood ES Brancaccio M . Generation of circadian rhythms in the suprachiasmatic nucleus. Nat Rev Neurosci. (2018) 19:453–69. doi: 10.1038/s41583-018-0026-z 29934559

[2] Hatori M Panda S . The emerging roles of melanopsin in behavioral adaptation to light. Trends Mol Med. (2010) 16:435–46. doi: 10.1016/j.molmed.2010.07.005 20810319 PMC2952704

[3] Ebling FJ . The role of glutamate in the photic regulation of the suprachiasmatic nucleus. Prog Neurobiol. (1996) 50:109–32. doi: 10.1016/s0301-0082(96)00032-9 8971980

[4] Sakamoto K Norona FE Alzate-Correa D Scarberry D Hoyt KR Obrietan K . Clock and light regulation of the CREB coactivator CRTC1 in the suprachiasmatic circadian clock. J Neurosci. (2013) 33:9021–7. doi: 10.1523/jneurosci.4202-12.2013 23699513 PMC3745023

[5] Laothamatas I Rasmussen ES Green CB Takahashi JS . Metabolic and chemical architecture of the mammalian circadian clock. Cell Chem Biol. (2023) 30:1033–52. doi: 10.1016/j.chembiol.2023.08.014 37708890 PMC10631358

[6] Takahashi JS . Transcriptional architecture of the mammalian circadian clock. Nat Rev Genet. (2017) 18:164–79. doi: 10.1038/nrg.2016.150 27990019 PMC5501165

[7] Narasimamurthy R Hunt SR Lu Y Fustin JM Okamura H Partch CL . CK1delta/epsilon protein kinase primes the PER2 circadian phosphoswitch. Proc Natl Acad Sci USA. (2018) 115:5986–91. doi: 10.1073/pnas.1721076115 29784789 PMC6003379

[8] Cho H Zhao X Hatori M Yu RT Barish GD Lam MT . Regulation of circadian behaviour and metabolism by REV-ERB-alpha and REV-ERB-beta. Nature. (2012) 485:123–7. doi: 10.1038/nature11048 22460952 PMC3367514

[9] Fagiani F Di Marino D Romagnoli A Travelli C Voltan D Di Cesare Mannelli L . Molecular regulations of circadian rhythm and implications for physiology and diseases. Signal Transduct Target Ther. (2022) 7:41. doi: 10.1038/s41392-022-00899-y 35136018 PMC8825842

[10] Peng F Li X Xiao F Zhao R Sun Z . Circadian clock, diurnal glucose metabolic rhythm, and dawn phenomenon. Trends Neurosci. (2022) 45:471–82. doi: 10.1016/j.tins.2022.03.010 35466006 PMC9117496

[11] Straif K Baan R Grosse Y Secretan B El Ghissassi F Bouvard V . Carcinogenicity of shift-work, painting, and fire-fighting. Lancet Oncol. (2007) 8:1065–6. doi: 10.1016/s1470-2045(07)70373-x 19271347

[12] Niu Y Zhu M . Circadian clock regulates immune checkpoint inhibitor efficacy. Acta Pharm Sin B. (2025) 15:1183–5. doi: 10.1016/j.apsb.2025.01.011 40177552 PMC11959908

[13] Wang C Zeng Q Gul ZM Wang S Pick R Cheng P . Circadian tumor infiltration and function of CD8(+) T cells dictate immunotherapy efficacy. Cell. (2024) 187:2690–702:e17. doi: 10.1016/j.cell.2024.04.015 38723627

[14] Tang H Wang Y Chlewicki LK Zhang Y Guo J Liang W . Facilitating T cell infiltration in tumor microenvironment overcomes resistance to PD-L1 blockade. Cancer Cell. (2016) 29:285–96. doi: 10.1016/j.ccell.2016.02.004 26977880 PMC4794755

[15] Leader AM Grout JA Maier BB Nabet BY Park MD Tabachnikova A . Single-cell analysis of human non-small cell lung cancer lesions refines tumor classification and patient stratification. Cancer Cell. (2021) 39:1594–609:e12. doi: 10.1016/j.ccell.2021.10.009 34767762 PMC8728963

[16] Fortin BM Pfeiffer SM Insua-Rodriguez J Alshetaiwi H Moshensky A Song WA . Circadian control of tumor immunosuppression affects efficacy of immune checkpoint blockade. Nat Immunol. (2024) 25:1257–69. doi: 10.1038/s41590-024-01859-0 38806707 PMC11374317

[17] Wang C Barnoud C Cenerenti M Sun M Caffa I Kizil B . Dendritic cells direct circadian anti-tumour immune responses. Nature. (2023) 614:136–43. doi: 10.1038/s41586-022-05605-0 36470303 PMC9891997

[18] Tian Y Zhang D . Biological clock and inflammatory bowel disease review: From the standpoint of the intestinal barrier. Gastroenterol Res Pract. (2022) 2022:2939921. doi: 10.1155/2022/2939921 35320972 PMC8938076

[19] Hua S Zhang Z Zhang Z Liu L Yu S Xiao Y . Genetic disruption of the circadian gene Bmal1 in the intestinal epithelium reduces colonic inflammation. EMBO Rep. (2025) 26:3138–61. doi: 10.1038/s44319-025-00464-y 40307620 PMC12187941

[20] Ruan W Li T Bang IH Lee J Deng W Ma X . BMAL1-HIF2A heterodimer modulates circadian variations of myocardial injury. Nature. (2025) 641:1017–28. doi: 10.1038/s41586-025-08898-z 40269168 PMC12095075

[21] Stokes K Cooke A Chang H Weaver DR Breault DT Karpowicz P . The circadian clock gene BMAL1 coordinates intestinal regeneration. Cell Mol Gastroenterol Hepatol. (2017) 4:95–114. doi: 10.1016/j.jcmgh.2017.03.011 28593182 PMC5453906

[22] Pu H Bailey LC Bauer LG Voronkov M Baxter M Huber KVM . Pharmacological targeting of BMAL1 modulates circadian and immune pathways. Nat Chem Biol. (2025) 21:736–45. doi: 10.1038/s41589-025-01863-x 40133642 PMC12037410

[23] Fellows RC Chun SK Larson N Fortin BM Mahieu AL Song WA . Disruption of the intestinal clock drives dysbiosis and impaired barrier function in colorectal cancer. Sci Adv. (2024) 10:eado1458. doi: 10.1126/sciadv.ado1458 39331712 PMC11430476

[24] Wu J Jing X Du Q Sun X Holgersson K Gao J . Disruption of the clock component Bmal1 in mice promotes cancer metastasis through the PAI-1-TGF-beta-myoCAF-dependent mechanism. Adv Sci (Weinh). (2023) 10:e2301505. doi: 10.1002/advs.202301505 37330661 PMC10460897

[25] Hou W . Role of TGFbeta-activated cancer-associated fibroblasts in the resistance to checkpoint blockade immunotherapy. Front Oncol. (2025) 15:1602452. doi: 10.3389/fonc.2025.1602452 40469190 PMC12133491

[26] Correa LH Correa R Farinasso CM de Sant'Ana Dourado LP Magalhaes KG . Adipocytes and macrophages interplay in the orchestration of tumor microenvironment: New implications in cancer progression. Front Immunol. (2017) 8:1129. doi: 10.3389/fimmu.2017.01129 28970834 PMC5609576

[27] Wang H Zhou F Qin W Yang Y Li X Liu R . Metabolic regulation of myeloid-derived suppressor cells in tumor immune microenvironment: Targets and therapeutic strategies. Theranostics. (2025) 15:2159–84. doi: 10.7150/thno.105276 39990210 PMC11840731

[28] Liu Z Selby CP Yang Y Lindsey-Boltz LA Cao X Eynullazada K . Circadian regulation of c-MYC in mice. Proc Natl Acad Sci USA. (2020) 117:21609–17. doi: 10.1073/pnas.2011225117 32817420 PMC7474601

[29] Deng J Jiang R Meng E Wu H . CXCL5: A coachman to drive cancer progression. Front Oncol. (2022) 12:944494. doi: 10.3389/fonc.2022.944494 35978824 PMC9376318

[30] Kramer ED Abrams SI . Granulocytic myeloid-derived suppressor cells as negative regulators of anticancer immunity. Front Immunol. (2020) 11:1963. doi: 10.3389/fimmu.2020.01963 32983128 PMC7481329

[31] Gabrilovich DI Nagaraj S . Myeloid-derived suppressor cells as regulators of the immune system. Nat Rev Immunol. (2009) 9:162–74. doi: 10.1038/nri2506 19197294 PMC2828349

[32] Liu JL Xu X Rixiati Y Wang CY Ni HL Chen WS . Dysfunctional circadian clock accelerates cancer metastasis by intestinal microbiota triggering accumulation of myeloid-derived suppressor cells. Cell Metab. (2024) 36:1320–34:e9. doi: 10.1016/j.cmet.2024.04.019 38838643

[33] Cabeza-Cabrerizo M Cardoso A Minutti CM Pereira da Costa M Reis e Sousa C . Dendritic cells revisited. Annu Rev Immunol. (2021) 39:131–66. doi: 10.1146/annurev-immunol-061020-053707 33481643

[34] du Bois H Heim TA Lund AW . Tumor-draining lymph nodes: At the crossroads of metastasis and immunity. Sci Immunol. (2021) 6:eabg3551. doi: 10.1126/sciimmunol.abg3551 34516744 PMC8628268

[35] Liu J Zhang X Cheng Y Cao X . Dendritic cell migration in inflammation and immunity. Cell Mol Immunol. (2021) 18:2461–71. doi: 10.1038/s41423-021-00726-4 34302064 PMC8298985

[36] Hanahan D . Hallmarks of cancer: New dimensions. Cancer Discov. (2022) 12:31–46. doi: 10.1158/2159-8290.cd-21-1059 35022204

[37] Kaymak I Watson MJ Oswald BM Ma S Johnson BK DeCamp LM . ACLY and ACSS2 link nutrient-dependent chromatin accessibility to CD8 T cell effector responses. J Exp Med. (2024) 221. doi: 10.1084/jem.20231820 39150482 PMC11329787

[38] Ling R Chen G Tang X Liu N Zhou Y Chen D . Acetyl-CoA synthetase 2(ACSS2): A review with a focus on metabolism and tumor development. Discov Oncol. (2022) 13:58. doi: 10.1007/s12672-022-00521-1 35798917 PMC9263018

[39] Wu H Zhao X Hochrein SM Eckstein M Gubert GF Knopper K . Mitochondrial dysfunction promotes the transition of precursor to terminally exhausted T cells through HIF-1alpha-mediated glycolytic reprogramming. Nat Commun. (2023) 14:6858. doi: 10.1038/s41467-023-42634-3 37891230 PMC10611730

[40] Dominguez M Brune B Namgaladze D . Exploring the role of ATP-citrate lyase in the immune system. Front Immunol. (2021) 12:632526. doi: 10.3389/fimmu.2021.632526 33679780 PMC7930476

[41] Lei X Zhang W Zhang D . Metabolic and epigenetic control of CD8(+) T cell exhaustion: The acetate-to-citrate switch. MedComm (2020). (2025) 6:e70307. doi: 10.1002/mco2.70307 40717903 PMC12290303

[42] Zhao J Lu Z Zhu J Duan Z Shen D Han H . Nervous system and cancer: Organ as the basic unit. iMetaMed. (2026) 2:e70036. doi: 10.1002/imm3.70036 41531421

[43] Zhao J Zhu J Yang Z Zhai Y Zhao C Lu Z . Extracellular vesicle-mediated O-GlcNAcase transfer drives neuronal necroptosis to facilitate gallbladder cancer perineural invasion. Cancer Res. (2026) 86:1392–413. doi: 10.1158/0008-5472.can-25-2237 41460724 PMC13012253

[44] Xuan W Khan F James CD Heimberger AB Lesniak MS Chen P . Circadian regulation of cancer cell and tumor microenvironment crosstalk. Trends Cell Biol. (2021) 31:940–50. doi: 10.1016/j.tcb.2021.06.008 34272133 PMC8526375

[45] Rahman S Wittine K Sedic M Markova-Car EP . Small molecules targeting biological clock; A novel prospective for anti-cancer drugs. Molecules. (2020) 25. doi: 10.3390/molecules25214937 33114496 PMC7663518

[46] Qu M . Molecular crosstalk between circadian clock and cancer and therapeutic implications. Front Nutr. (2023) 10:1143001. doi: 10.3389/fnut.2023.1143001 36937362 PMC10017454

[47] Ishimaru K Nakajima S Yu G Nakamura Y Nakao A . The putatively specific synthetic REV-ERB agonist SR9009 inhibits IgE- and IL-33-mediated mast cell activation independently of the circadian clock. Int J Mol Sci. (2019) 20. doi: 10.3390/ijms20246320 31847374 PMC6941044

[48] Sulli G Rommel A Wang X Kolar MJ Puca F Saghatelian A . Pharmacological activation of REV-ERBs is lethal in cancer and oncogene-induced senescence. Nature. (2018) 553:351–5. doi: 10.1038/nature25170 29320480 PMC5924733

[49] Scheiermann C Kunisaki Y Frenette PS . Circadian control of the immune system. Nat Rev Immunol. (2013) 13:190–8. doi: 10.1038/nri3386 23391992 PMC4090048

[50] Noel R Song X Shin Y Banerjee S Kojetin D Lin L . Synthesis and SAR of tetrahydroisoquinolines as Rev-erbalpha agonists. Bioorg Med Chem Lett. (2012) 22:3739–42. doi: 10.1016/j.bmcl.2012.04.023 22560469 PMC3488456

[51] Wolff SEC Wang XL Jiao H Sun J Kalsbeek A Yi CX . The effect of Rev-erbalpha agonist SR9011 on the immune response and cell metabolism of microglia. Front Immunol. (2020) 11:550145. doi: 10.3389/fimmu.2020.550145 33101272 PMC7546349

[52] Ribeiro RFN Cavadas C Silva MMC . Small-molecule modulators of the circadian clock: Pharmacological potentials in circadian-related diseases. Drug Discov Today. (2021) 26:1620–41. doi: 10.1016/j.drudis.2021.03.015 33781946

[53] Jeong YU Jin HE Lim HY Choi G Joo H Kang B . Development of non-ethoxypropanoic acid type cryptochrome inhibitors with circadian molecular clock-enhancing activity by bioisosteric replacement. Pharm (Basel). (2021) 14. doi: 10.3390/ph14060496 34073760 PMC8225008

[54] de Vries R Doveston RG Meijer FA Brunsveld L . Elucidation of an allosteric mode of action for a thienopyrazole RORgammat inverse agonist. ChemMedChem. (2020) 15:561–5. doi: 10.1002/cmdc.202000044 32053744 PMC7187189

[55] Lellupitiyage Don SS Robertson KL Lin HH Labriola C Harrington ME Taylor SR . Nobiletin affects circadian rhythms and oncogenic characteristics in a cell-dependent manner. PloS One. (2020) 15:e0236315. doi: 10.1371/journal.pone.0236315 32706791 PMC7380617

[56] Suzuki S Nakamura T Saito R Terauchi Y Kawai K Takimoto-Kamimura M . Structural change of retinoic-acid receptor-related orphan receptor induced by binding of inverse-agonist: Molecular dynamics and ab initio molecular orbital simulations. Comput Struct Biotechnol J. (2020) 18:1676–85. doi: 10.1016/j.csbj.2020.06.034 32670507 PMC7338990

[57] Ballesta A Innominato PF Dallmann R Rand DA Levi FA . Systems chronotherapeutics. Pharmacol Rev. (2017) 69:161–99. doi: 10.1124/pr.116.013441 28351863 PMC5394920

[58] Zeeshan M Hu J Mao CX Danish A Xiong Y Irshad MS . Nanomaterial-enabled drug delivery systems for circadian medicine: bridging direct rhythm modulation and chronotherapy. RSC Adv. (2025) 15:31981–2008. doi: 10.1039/d5ra04137f 40917604 PMC12412127

[59] Wang C Ye Y Hu Q Bellotti A Gu Z . Tailoring biomaterials for cancer immunotherapy: emerging trends and future outlook. Adv Mater. (2017) 29. doi: 10.1002/adma.201606036 28556553

[60] Ozturk N Ozturk D Kavakli IH Okyar A . Molecular aspects of circadian pharmacology and relevance for cancer chronotherapy. Int J Mol Sci. (2017) 18. doi: 10.3390/ijms18102168 29039812 PMC5666849

[61] Guan F Wang R Yi Z Luo P Liu W Xie Y . Tissue macrophages: origin, heterogenity, biological functions, diseases and therapeutic targets. Signal Transduct Target Ther. (2025) 10:93. doi: 10.1038/s41392-025-02124-y 40055311 PMC11889221

[62] Nilsson A Peters JM Meimetis N Bryson B Lauffenburger DA . Artificial neural networks enable genome-scale simulations of intracellular signaling. Nat Commun. (2022) 13:3069. doi: 10.1038/s41467-022-30684-y 35654811 PMC9163072

[63] Sun X Qin L Liang X Wang D . Circadian rhythm in immunotherapy and cellular therapy: impacts on the tumor microenvironment. Acta Biochim Biophys Sin (Shanghai). (2026) 58:90–105. doi: 10.3724/abbs.2025203 41261920 PMC12862608

[64] Cash E Sephton S Woolley C Elbehi AM R IA Ekine-Afolabi B . The role of the circadian clock in cancer hallmark acquisition and immune-based cancer therapeutics. J Exp Clin Cancer Res. (2021) 40:119. doi: 10.1186/s13046-021-01919-5 33794967 PMC8017624

[65] Zhang Z Zeng P Gao W Zhou Q Feng T Tian X . Circadian clock: a regulator of the immunity in cancer. Cell Commun Signal. (2021) 19:37. doi: 10.1186/s12964-021-00721-2 33752691 PMC7986390

[66] Lyons PG Gill E Kumar P Beasley M Park-Egan B Lokhandwala ZA . Diurnal rhythm in chimeric antigen receptor T cell effectiveness in an observational study of 715 patients. JCI Insight. (2026) 11. doi: 10.1172/jci.insight.201159 41411053 PMC12892897

[67] Zerbib J Bloomberg A Ben-David U . Targeting vulnerabilities of aneuploid cells for cancer therapy. Trends Cancer. (2025) 11:642–64. doi: 10.1016/j.trecan.2025.04.005 40368673

[68] Robinson AS Bennett S Sato S . When clocks go rogue: circadian rhythms and the rise of cancer. J Biol Rhythms. (2026) 41:155–77. doi: 10.1177/07487304251391270 41520235

[69] Balachandran DD Bashoura L Sheshadri A Manzullo E Faiz SA . The impact of immunotherapy on sleep and circadian rhythms in patients with cancer. Front Oncol. (2023) 13:1295267. doi: 10.3389/fonc.2023.1295267 38090501 PMC10711041

[70] Karaboue A Innominato PF Wreglesworth NI Duchemann B Adam R Levi FA . Why does circadian timing of administration matter for immune checkpoint inhibitors' efficacy? Br J Cancer. (2024) 131:783–96. doi: 10.1038/s41416-024-02704-9 PMC11369086 38834742

[71] Pick R Wang C Zeng Q Gul ZM Scheiermann C . Circadian rhythms in anticancer immunity: mechanisms and treatment opportunities. Annu Rev Immunol. (2024) 42:83–102. doi: 10.1146/annurev-immunol-090122-050842 38941606

[72] Fey RM Billo A Clister T Doan KL Berry EG Tibbitts DC . Personalization of cancer treatment: exploring the role of chronotherapy in immune checkpoint inhibitor efficacy. Cancers (Basel). (2025) 17. doi: 10.3390/cancers17050732 40075580 PMC11899640

[73] Van Loocke P Beuselinck B Meulemans J Nuyts S Willaert R Di Santo D . Impact of time-of-day administration of immune checkpoint inhibitors on survival outcomes in patients with recurrent/metastatic head and neck cancer (R/M HNSCC): a retrospective cohort study. Oral Oncol. (2025) 171:107772. doi: 10.1016/j.oraloncology.2025.107772 41202429

[74] Shim J Fleisch E Barata F . Circadian rhythm analysis using wearable-based accelerometry as a digital biomarker of aging and healthspan. NPJ Digit Med. (2024) 7:146. doi: 10.1038/s41746-024-01111-x 38834756 PMC11150246

[75] De Domenico M Allegri L Caldarelli G d'Andrea V Di Camillo B Rocha LM . Challenges and opportunities for digital twins in precision medicine from a complex systems perspective. NPJ Digit Med. (2025) 8:37. doi: 10.1038/s41746-024-01402-3 39825012 PMC11742446

[76] Levi FA Okyar A Hadadi E Innominato PF Ballesta A . Circadian regulation of drug responses: toward sex-specific and personalized chronotherapy. Annu Rev Pharmacol Toxicol. (2024) 64:89–114. doi: 10.1146/annurev-pharmtox-051920-095416 37722720

[77] Zhao Z Wu S Wang T Zhao Y . Gut microbiota circadian rhythms: a key regulator of immunometabolic homeostasis. Acta Biochim Biophys Sin (Shanghai). (2026) 58:106–19. doi: 10.3724/abbs.2025220 41311218 PMC12862605

[78] Butler CT Rodgers AM Curtis AM Donnelly RF . Chrono-tailored drug delivery systems: recent advances and future directions. Drug Delivery Transl Res. (2024) 14:1756–75. doi: 10.1007/s13346-024-01539-4 38416386 PMC11153310

